# Risk Shifting in Gastroenterology

**DOI:** 10.1016/j.gastha.2022.02.019

**Published:** 2022-04-29

**Authors:** Amnon Sonnenberg, Gennadiy Bakis

**Affiliations:** The Portland VA Medical Center, Oregon Health & Science University, Portland, Oregon; Division of Gastroenterology and Hepatology, Oregon Health & Science University, Portland, Oregon; Division of Gastroenterology and Hepatology, Oregon Health & Science University, Portland, Oregon

## The Occurrence of Risk Shifting

Gastroenterologists frequently encounter challenging medical situations with high risks for adverse events associated with endoscopic interventions or medical treatment plans. For instance, dilation of esophageal strictures and placement of esophageal stents can result in perforation or stent migration. Trying to achieve hemostasis of gastrointestinal bleeding may be unsuccessful or result in perforation.^1^, ^2^, ^3^ Snare polypectomy or endoscopic mucosal resection can similarly result in bleeding and perforation.^4^^,^^5^ Innumerable other examples exist.^6^^,^^7^ Gastroenterologists and physicians in general try avoiding adverse outcomes because they do not want to inflict harm to their patients. They may also be concerned about litigation, the hassle of possible law suits, and the impact such adverse events may have on their professional reputation.^8^, ^9^, ^10^, ^11^ One strategy to avoid incurring adverse events is to refer the patient to a colleague, surgeon, or interventional radiologist and let them deal with the problem at hand. Sometimes, one can call upon a colleague to share in the decision-making or cosign the chart note and have them become involved and assume partial responsibility for any future outcomes. On one hand side, such risk shifting or sharing is beneficial from the physicians’ perspective, as it reduces their risks and corresponding costs.^12^^,^^13^ On the other hand, risk shifting may not be beneficial from the patients’ perspective. Their treatment may become delayed, and their discomfort may be prolonged. Patients may be forced to undergo the same procedure twice or be subjected to additional and more invasive procedures. Lastly, the alternative procedures may provide, compared with the initial option, a lesser alternative with a less favorable outcome, more discomfort, and potentially even higher medical risks.

## Examples of Risk Shifting

Few more examples may serve to further illustrate the points from above. The hospital stay of a patient with acute pancreatitis became unduly prolonged because the interventional endoscopist was hesitant to drain the necrotic collection, hoping that the problem would eventually resolve on its own or be dealt with by someone else at a later point in time. After a failed initial biliary cannulation by endoscopic retrograde cholangio-pancreatography, a patient with biliary stricture was referred to interventional radiology and received an inconvenient percutaneous drainage because the endoscopist was overly concerned about the risks associated with utilizing a needle knife or endoscopic ultrasound-guided biliary access. Yet another patient with a large but benign cecal polyp was referred to surgery for right hemicolectomy rather than undergoing an endoscopic mucosal resection. Sometimes, patients are being referred to interventional radiology for percutaneous gastrostomy or hemostasis of bleeding ulcers before exhausting the corresponding endoscopic means of treatment. Other examples abound.

## The Gray Zone of Risk Shifting

The clinical scenarios from above need to be contrasted with the majority of instances where referrals to a colleague with more expertise in handling a complex diagnostic or therapeutic problem are fully justified. In general, physicians are specifically advised to refer their difficult patients to specialists sooner rather than later to avoid future legal troubles.^14^ However, there is a gray zone that stretches between justified and unjustified referrals and makes it easy for referring physicians to hide their motivations when deferring potentially risky medical decisions. Unskilled but conscientious gastroenterologists, who know their own limitations, may benefit their patients by referring them to other endoscopists even for dealing with minor issues. Such occurrence reflects on the general paradox of the inept or timid physician, who serves his patients best by referring them elsewhere. A proclivity to refer patients to other professionals for minor medical problems has also been recognized as one of the signs that indicate a general decline among aging physician in fulfilling their professional duties.^15^^,^^16^ Besides the inherent difficulties in separating necessary from unnecessary consults, there are usually few means or little time available to tease out the frivolous from the justified consults. Out of professional courtesy, sense of duty, and avoidance of patient abandonment, in general, all referrals are being accepted and dealt with. Such response to all referrals also reflects on the downside of a highly litigious medical-legal environment where all real or imagined errors of omission are being severely disciplined—In the following section, we provide a short microeconomic analysis of the driving forces behind risk shifting. A reader less interested in such quantitative analysis may skip this section without much loss in a train of thought.

## The Economics of Risk Shifting

The driving forces for risk shifting are easy to phrase in economic terms. In the following context, the terms risks and costs will be used synonymously, and besides financial expenditures, costs will be thought to also include discomfort, pain, fear, and possible death. From a patient perspective, the costs before and after risk shifting will be *C*_*1*_ and *C*_*2*_, respectively. The analysis is focused on unjustified referrals which benefit only the referring physician and increase the patient’s own costs, that is, *C*_*2*_ *> C*_*1*_. The physician’s costs before and after risk shifting are expressed as a fraction of the patients' initial costs, that is *a*_*1*_*‧C*_*1*_ and *a*_*2*_*‧C*_*1*_, respectively, where *a* denotes a cost ratio. (Individual physicians and different scenarios may be characterized by different cost ratios.) It is assumed that the physician’s costs after risk shifting will decrease, that is, *a*_*1*_ *> a*_*2*_. There is an incentive for a physician to shift cost, as long as the initial overall costs are higher than those following risk shifting, that is,

$$C_{1}+a_{1}\cdot C_{1}>C_{2}+a_{2}\cdot C_{1}$$

Few simple algebraic transformations lead to

$$1+\left(a_{1}-a_{2}\right)>C_{2}/C_{1}$$

If all risk can be fully transferred to another physician and *a*_*2*_ *= 0*, the last equation turns into the following:

$$1+a_{1}>C_{2}/C_{1}$$

The last equation compares 2 cost ratios. On the left side is the ratio of physician-to-patient costs. The right side contains the ratio of patient costs before and after risk shifting. The linear relationship between the 2 ratios indicates that physicians who are more concerned about their own risk are also more inclined to shift their risk elsewhere. They continue to shift as long as their own perceived costs exceed their patients’ increase in costs, arising from having their care being transferred elsewhere. Assuming, for instance, that a physician perceives his/her own personal risk associated with the planned intervention to be *a*_*1*_ = 0.3 compared with the patient’s overall costs, then the patient’s own costs after risk shifting would need to increase 1.3-fold, before risk shifting would no longer be considered feasible. If the physician personal risk associated with the planned intervention is 2-fold higher than the patient’s costs, then the patient’s own costs after risk shifting would need to increase 3-fold, before risk shifting would no longer be considered feasible. In instances of risk sharing, *a*_*2*_ will not drop to zero, as the first physician would still retain some or most of the initial risk, with *a*_*2*_ *≈ a*_*1*_. In such instances, *C*_*2*_ would also need to be fairly close to *C*_*1*_, with *C*_*2*_ *≈ C*_*1*_. For example, such scenarios pertain to instances where another physician is made a cosigner on a chart note or is being pulled otherwise into the process of decision-making without such means of risk sharing affecting the patient’s actual costs.

## Outlook and Conclusions

It again needs to be emphasized that the scenarios underlying the analysis from above do not apply to many situations, where the knowledge base or skillset of an individual physician has been exhausted and the added input from a colleague or another subspecialist would advance disease management. In contrast to such necessary and productive interactions, the practice of risk shifting abuses the common channels of collaboration and consultation for offloading potentially difficult cases that still lie within the expected performance range of the referring physician. Such behavior reflects on a refusal to do one’s work, accept challenging cases, and lean on others to act on one’s behalf. Risk shifting disguises itself as a collegial interaction among physicians, when in reality, it only serves to reduce one’s own risks and costs with no benefit to the patient. Its primary aim is to avoid personal responsibility and minimize the feared risk of adverse outcomes. This behavioral pattern also ignores the inconvenience, risks, and costs that it imposes on one’s colleagues from gastroenterology and other subspecialties. It thus renders medicine overall less effective and more costly. The underlying problem is caused by a distorted medical-legal system and its misplaced financial incentives, which promote an artificial conflict of interests between physicians and their patients. Eventually, this dilemma could possibly be resolved if the patients’ interests and well-being became better separated from those of their physicians, and physicians would need to be less concerned about their own interests when making medical decisions.
